# Exploration of social factors associated to maternal deaths due to haemorrhage and convulsions: Analysis of 28 social autopsies in rural Bangladesh

**DOI:** 10.1186/s12913-016-1912-6

**Published:** 2016-11-15

**Authors:** Animesh Biswas, M. A. Halim, Koustuv Dalal, Fazlur Rahman

**Affiliations:** 1 Department of Public Health Science, School of Health Sciences, Örebro University, Örebro, Sweden; 2 Reproductive and Child Health Unit, Centre for Injury Prevention and Research, Bangladesh (CIPRB), House B 162, Road 23, New DOHS, Mohakahlai, Dhaka 1206 Bangladesh

**Keywords:** Maternal death, Social autopsy, Death review, Rural community, Bangladesh

## Abstract

**Background:**

Social autopsy is an innovative approach to explore social barriers and factors associated to a death in the community. The process also sensitize the community people to avert future deaths. Social autopsy has been introduced in maternal deaths in Bangladesh first time in 2010. This study is to identify the social factors in the rural community associated to maternal deaths. It also looks at how the community responses in social autopsy intervention to prevent future maternal deaths.

**Methods:**

The study was conducted in the Thakurgaon district of Bangladesh in 2010. We have purposively selected 28 social autopsy cases of which maternal deaths occurred due to either haemorrhage or due to convulsions. The autopsy was conducted by the Government health and family planning first line field supervisors in rural community. Family members and neighbours of the deceased participated in each autopsy and provided their comments and responses.

**Results:**

A number of social factors including delivery conducted by the untrained birth attendant or family members, delays in understanding about maternal complications, delays in decision making to transfer the mother, lack of proper knowledge, education and traditional myth influences the maternal deaths. The community identified their own problems, shared within them and decide upon rectify themselves for future death prevention.

**Conclusions:**

Social autopsy is a useful tools to identify social community within the community by discussing the factors that took place during a maternal death. The process supports villagers to think and change their behavioural patterns and commit towards preventing such deaths in the future.

## Background

Bangladesh has made a substantial improvement in maternal death reduction in the past decades. Maternal deaths have been reduced to 194/100000 live births in 2010 compared to 322/1000000 in a 2001 survey report [1–4]. Recent data shows that maternal deaths have been decreased to 170/100,000 live births [5, 6]. Majority of the mothers are still dying in Bangladesh is due to post-partum haemorrhage and pre-eclampsia/eclampsia [5, 6]. A large proportion of deliveries are conducted at home by untrained birth attendants or relatives [5, 7]. Maternal and neonatal death review (MNDR) has been in operation in Bangladesh since 2010 and was first implemented in one district of Bangladesh named Thakurgaon [8]. In the death review system follows notification of each of the maternal deaths from the community followed by verbal autopsy to identify medical causes of death. Verbal autopsy also helped to provide understanding the context and the details surrounding the death event [8, 9]. Verbal autopsy findings of maternal deaths in MNDR revealed that the majority of mothers are dying of complications in the health facility or on the way towards the health facility as a result of poor community decision making and transportation delays, these often place the mother in crisis [5]. Social autopsy in MNDR has been introduced as social intervention in the community to explore the social, behavioural and medical causes, including errors and barriers in the community that have contributing towards a death [10–13]. Social autopsy (SA) is an interactive discussion meeting (courtyard meeting) with the neighbours of deceased to share and explore the circumstances behind the death and how it could have been averted [14]. Social autopsy also acts to prompt the minds of the people affected by the death to think and discuss positively what could have been done differently to prevent the death [2, 14]. The meeting also seeks a commitment from the community to combat such events in the future.

This study aims to identify the social factors in the rural community associated to maternal deaths. The study also looks at how the community responses in social autopsy intervention to prevent future maternal deaths.

## Methods

The study was performed in Thakurgaon district of Bangladesh which is situated on the northern region of Bangladesh, approximately 450 km from the capital Dhaka. The district has a population of approximately 1.4 million, distributed in five sub-districts (upazila). The study was conducted in between January to December in 2010. A total of 59 maternal deaths were notified by the government health and family planning staff though the MNDR system in the district [9]. The study has recruited 28 maternal deaths cases in the district out of total 59 reported maternal deaths in 2010. Inclusion of the cases was those who had either convulsions or haemorrhage caused maternal deaths. For inclusion of the cases, verbal autopsy data of all maternal deaths were reviewed to get the selected cases. For each of the selected death cases, social autopsy was performed totaling 28 SAs. In the government health system, the first line field supervisor from health and family planning department (Health Inspector, Assistant Health Inspector and Family Planning Inspector) of assigned area was responsible to facilitate the social autopsy session.

Social autopsy has performed in a premises in the rural community near to the deceased home. The autopsy meeting surrounded by 20–50 neighbours of different age group and gender. The facilitator do dialog with the community and describe the purpose of doing social autopsy and utilizing of findings. A note taker participated with the facilitator kept the note of the meeting following a guideline for record keeping of the meeting. Anonymity and confidentiality was correctly maintained with respondents in the study willingly participating in the event. During the process participants had the ability to respond freely or leave the process at any point. The participants were guided to discuss on social factors, barriers related to the death. They were also disused on what possible solutions or steps to be taken by the community to prevent future deaths.

### Process of social autopsy data collection

Neighbors of the deceased household were the participants of the social autopsy, and approximately 20–50 contributors were present at each social autopsy meeting. Targeted participants for the meeting included; head of the households, women of reproductive age, representatives from adolescent groups, elders, and elite persons of the village (Imam, school teachers, religious leaders, Chairperson and elective members). The autopsy was usually conducted during the afternoon or in the early morning. This allowed male participants, mostly the decision makers of the family, to attend the meeting. The duration of each social autopsy meeting varied from 30 min to 1 h.

### Data retrieve

Each meeting was chaired by a representative from the community. The facilitator (Health/Family Planning Inspector) started the meeting with the permission of Chairperson. The facilitator initiated discussion by described the event and illustrating the circumstances before the mother died. The facilitator asked some key points of the autopsy participants on what mistakes or errors were made from a community perspective in the death and what remedial action would need to be taken in future to prevent such situations. Those questions prompt and help the community in exploring social errors behind the death. When the participants started expressing their opinions, the facilitator encouraged further discussion. Finally, the community decides on some preventable social factors to avert such types of deaths and makes a commitment to avoid such complications during the mother’s pregnancy and to promote safe delivery. At the end of the discussion the facilitator showed pictorial communication materials containing messages on maternal danger signs, birth planning, antenatal care and importance of institutional delivery. Hard copies of the notes were kept after each of the social autopsy for data analysis.

### Data analysis

A descriptive analysis of the general characteristics of the mother was performed in Microsoft Excel. Qualitative information was obtained from the hand written notes taken and translated into English from the local language Bengali. Two experienced bi-lingual researchers have independently checked the translation. Those notes were read and re-read to find out what social stigma were discussed, how community behaved on decision making for future death prevention. Open code followed by selective coded data was done by two experienced researchers. Theme were identified from the text and it was manually analyzed by an anthropologist as per theme.

## Results

We analyzed social autopsies of 12 bleeding (haemorrhage), including antepartum and post-partum cases, and another 16 maternal death cases with convulsion (pre-eclampsia/eclampsia). The mean age of the mother was 25 with the minimum 16 and maximum 45 years of age. Majority of the mothers were aged between 18–29 years (60.7 %). Thirty-six percent of mothers had no formal education whereas, 25.1 % had received education up to five grade. The mean age of marriage of the mother was found to be 17 with a minimum age of 13 and a maximum of 25. Over 78 % of the mothers married before 18 years of age. Around 86 % of the mothers had full term pregnancy and 75 % of them had a livebirths as delivery outcome [Table 1].

**Table 1:** Mothers’ characteristics

Mother’s age	In percentage
< 18 years	14.3
18–29 years	60.7
30 years and above	25
Mother’s education	
Up to five grade	25.1
Six to ten grade	32.1
11 grade completed and above	7.2
No education	35.7
Age of mother at the time of marriage	
< 18 years	78.6
18 years and above	21.4
Duration of pregnancy	
Eight months of pregnancy	14.3
Nine months full term pregnancy	85.7
Outcome of the delivery	
Live birth	75
Stillbirth	14.3
Not delivered	10.7

**Fig. 1:**
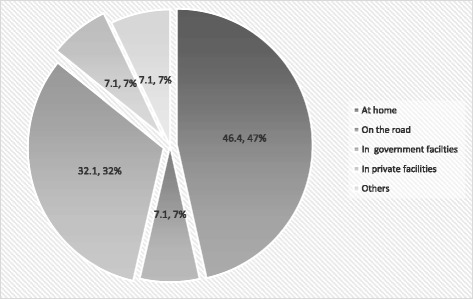
Place of Death of Mothers

**Fig. 2:**
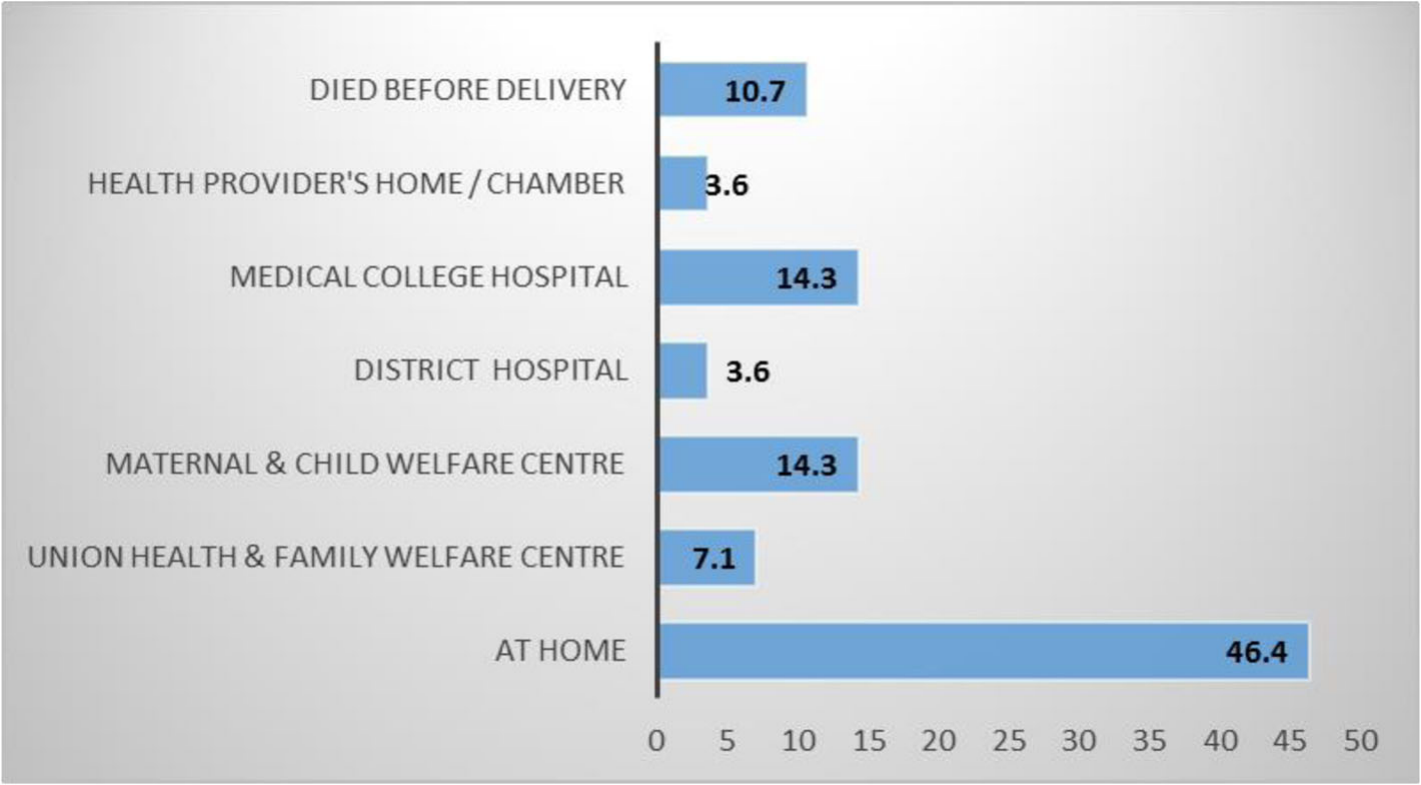
Place of Delivery of Mothers

89.3 % of mothers (*n* = 25) died after the delivery with the remainder dying during pregnancy (10.7 %). The majority of mothers died at home (46.4 %) [Fig. 1]. Mothers who died during home delivery were found in 46.4 % cases [Fig. 2] and delivery conducted by an untrained birth attendant was approximately 36 %.

A total number of 761 participants from the community participated in the social autopsy meetings. Of these 226 were male and 535 were female. During social autopsies where the mother had died due to convulsion and bleeding, it was found in convulsion cases that the mother had high blood pressure and swelling of limbs and face. A history of convulsion and unconsciousness was also reported.

### Key findings related to convulsion

Majority of the mothers were found with high blood pressure, blurring of vision, swelling of face and leg. However, family was not aware of those as maternal complications. Mothers were also had lack of adequate antenatal care during pregnancy. Family also had delayed in decision making to seek treatment before death. Moreover, families had also had superstition about convulsions of mothers. The participants in the social; autopsies expressed importance of quality antennal care and ensure delivery at the facility [Table 2].

**Table 2:** Maternal deaths as a consequence of convulsion

Case	Key findings on the death issue discussed	Social errors perceived	Responses of the community
01	High blood pressure (HBP) with sudden unconsciousness. Mother didn’t receive regular ANC during pregnancy. Labour pains started at 4 am. The village doctor was called and came at 6 am at morning. The Doctor tried to conduct delivery for 2:30 h, then finally referred to facility. Vehicle was not available and family had no plan as to where to go.	Regular antenatal checkup was not received. Village doctor was called for delivery.	All pregnant mothers will receive ANC in future. Family will prepare birth planning. Mothers will deliver babies at the health facility.
02	At nine months of pregnancy the patient had sudden convulsion and started to bleed. Mother was then brought to hospital but died before treatment could commence.	Family was not aware of mother’s underlying illness	Regular ANC must be ensured. Community need to be aware of danger signs during pregnancy.
03	Had blurring of vision, swelling of leg and face. Mother didn’t receive treatment from health professionals but rather took some medicine from local traditional healer.	Family didn’t make plans to go to medical facility for better management	Community will become aware of sending a mother with complications to hospital for care & delivery.
04	Mother was in term. Prolonged labour was for 24 h with convulsion history during pregnancy. Family delayed and as family were undecided where to go the birth took place at home.	Mother was not taken to hospital at right time due to decision delay.	Community will encourage mother to deliver baby at the facility. Danger signs during pregnancy must be known.
05	Had HBP, fever, blurring of vision, unconsciousness. Mother hadn’t received regular ANC because of superstition in the family and also in community about hospital services and delivery.	Mother had early marriage, inadequate ANC. Poor perception of health facility within the community.	All pregnant mother will receive ANC in future. Community will deliver baby at the facility. Community will not encourage early marriage.
06	Had HBP, unable to be treated due to lack of money, Village doctor was called during delivery, he failed and finally referred mother to hospital. The mother died after just after baby delivered.	Mother was not taken to hospital at right time due to delayed referral.	In future all pregnant mothers will receive ANC.
07	Had HBP, decision was delayed to go to facility	Mother was not taken to hospital at right time due to decision delay.	In future all pregnant mothers will receive ANC.
08	Had HBP, blurring of vision. During ANC she was advised to come to hospital at the time of delivery but she didn’t. Delivery took place at home and she died soon after.	The mother was not taken to the health facility for delivery	In future all pregnant mothers will receive ANC. Community will ensure baby is delivered at the facility.
09	Had swelling of limbs and HBP. Mother hadn’t gone for regular ANC visit. During delivery family called a traditional birth attendant who was unable to deliver.	Mother was not regularly treat by a medical doctor.	All pregnant mothers will receive ANC. Community will ensure baby is delivered at the facility.
10	Had high BP, blurring of vision and unconsciousness. Patient was cared for at home by untrained birth attendant.	Family didn’t take decision to go hospital, delivery performed by untrained person.	All pregnant mothers will receive ANC in future. Family will undertake birth planning and for the delivery of the baby at hospital.
11	At eight months, had vomiting and HBP. Mother was admitted to Upazila health complex but was unable to be treated there, so referred to the district hospital where she died.	Delay in decisions making by the family	Community should know the maternal danger signs and plan to have delivery at health facility.
12	Had convulsion HBP. ANC received three times. Traditional birth attendant was called for delivery. Delay in decision making to take the mother at hospital.	Delay in decision making but finally referred the patient to facility	All pregnant mothers will receive ANC. Family will prepare birth planning.
13	Had severe headache and vomiting. Convulsion began at midnight when labour pain started. Family waited till morning and it took four hours to arrive in upazila health complex. Unable to be treated there, she was referred to district hospital but died within 30 min after delivering a still birth.	Delay in decisions making, lack of transport facility and didn’t receive regular ANC.	All pregnant mothers will receive ANC. Family will prepare birth planning.
14	Had fever with swelling of limbs. Mother had low weight due to family member not providing Mother with sufficient food. Family thought the coming baby would be large in size and normal delivery could not take place. Villagers had a misperception about care at the health facility.	Family had misperception and had not prepared for the delivery. Mother did not receive any ANC	All pregnant mothers will receive ANC. Family will prepare birth planning.
15	Had leg swelling, convulsion before delivery and HBP. Normal delivery conducted at facility. After a few hours of the delivery mother was returning back home when convulsions developed whilst travelling. Mother died within five minutes after arriving home.	Mother did not attend regular checkup during pregnancy.	Regular ANC must be ensured. Danger signs during pregnancy need to be known.
16	Mother had severe convulsion before delivery. Delivery conducted by the TBA at home. Mother died just after the delivery.	No ANC was received and family was not aware about maternal complications.	Regular ANC must be ensured. Community will deliver baby at the facility.

### Key findings related to bleeding

In regard to bleeding, casual analysis found that the majority of mothers had bleeding after delivery when the placenta was retained after an obstructed labour. In the majority of cases where delivery was performed at home, it was found that complications to the mother could not be managed by the untrained birth attendant. Of those who had finally decided at the last moment to be referred to a hospital, the mother died either on the road or on arrival at the health facility. Community representatives, including family of the deceased, identified a number of barriers, mostly related to not taking the mother to the health facility, lack of adequate antenatal care (ANC), delays in referral, and delivery conducted by poorly trained attendants. However, the community stated that in future they would ensure ANC of the mother, strengthen the referral system, ensure delivery at the health facility and promote sound birth planning [Table 3].

**Table 3:** Maternal deaths occurred due to consequences of bleeding

Case	Key findings on the death issue discussed	Social errors perceived	Responses of the community
01	During delivery family members called village doctor and traditional birth attendant. Delivery conducted after 17 h of labour pain. After delivery placenta was retained and bleeding started. Patient referred to district hospital. Mother died just after arrive at hospital.	Mother didn’t receive any ANC and family didn’t prepare any birth planning.	Each expectant mother should receive regular ANC, prepare a birth planning and ensure delivery at a health facility.
02	The mother delivered a live birth by traditional birth attendant but the placenta was not removed and started bleeding. Family called village doctor and he gave some injections. After a while he referred the patient to the facility, but the patient died before arrival at hospital.	Family delayed in decision making and didn’t baby not delivered at health facility.	Each mother needs to deliver baby at health facility by trained provider.
03	The mother delivered a live birth by traditional birth attendant, profuse bleeding just after delivery. Family called village doctor and he gave some saline and injections. Life was lost within 30 min after treatment initiated at home.	Didn’t have birth planning and baby delivered by untrained birth attendant at home.	Each mother needs to deliver their baby at health facility by trained provider and prepare birth planning.
04	When labour pain started, family members called village doctor and a traditional birth attendant. Profuse bleeding started just after delivery. It took 4 h at home to take the decision to take the patient to the health facility. The mother died just after 30 min of arrival at hospital.	Family didn’t take decision at right time and there was a lack of transport to carry the patient.	Prepare birth planning and ensure health facility delivery. Make the community more aware about maternal danger signs.
05	The mother delivered a live birth by traditional birth attendant at home but placenta retained and started bleeding, TBA tried for two to three hours but the mother died on the way towards health facility.	Family delayed their decision making for seven hours.	Mother should receive regular ANC, prepare birth planning and ensure health facility delivery.
06	The mother delivered a live birth by mother in law and sister in law at home. Placenta was retained and bleeding started. Relatives failed to manage the bleeding, called the village doctor but the patient died at home without any treatment	Family made mistake of trying to deliver at home and delayed for hours the decision to seek help.	Mother should receive regular ANC, prepare a birth planning and ensure facility delivery. More community awareness of maternal complications.
07	Traditional birth attendant delivered baby at home, bleeding started just after delivery. Mother become unconscious after some time and TBA couldn’t manage complications. It was dark night and no vehicle was available to transport the patient to the health facility. Mother died at home.	Family delayed making the decision and there was a lack of transport to carry the patient at night.	Mother should receive regular ANC, prepare a Birth planning and ensure health facility delivery.
08	The mother delivered a live birth by traditional birth attendant at home in presence of village doctor but bleeding was profuse after delivery. Family unable to carry patient at right time and due to the lack of vehicle at night it took four hours to bring the patient to the health facility.	Family delayed making the decision and there was a lack of transport to carry the patient at night.	Mother should receive regular ANC, prepare birth planning and ensure facility delivery
09	The mother delivered a live birth by traditional birth attendant at home. After 9 days she developed convulsion with bleeding. Mother admitted to hospital and treated, the family took the mother back to a village doctor’s home. Mother died at village doctor’s home	Family made wrong decisions and were not aware of maternal complications.	Mother should receive regular ANC, prepare birth planning and ensure facility delivery.
10	Mother delivered a baby by an untrained birth attendant at home and severe bleeding started after delivery due to retain placenta.	Mother delivered a baby by an untrained birth attendant at home and severe bleeding started after delivery due to retain placenta.	Each mother should receive regular ANC, prepare birth planning and ensure facility delivery.
11	Mother didn’t seek treatment due to lack of enough money. The family also had misconception of hospital treatment. Bleeding started after delivery of a still birth and mother died at home without any treatment.	Mother not treated at health facility and didn’t receive regular ANC	Each mother should receive regular ANC, prepare birth planning and ensure facility delivery.
12	Mother delivered a live birth by traditional birth attendant at home after a prolonged labour, bleeding started just after delivery. Family was not prepared to carry the patient to health facility due to lack of vehicle and money.	Lack of birth planning and delivery conducted at home.	Each mother should receive regular ANC, prepare birth planning and ensure facility delivery.

## Discussions

The study conducted social autopsy focusing maternal death due to haemorrhage and convulsion, mostly after the delivery. The case discussion note presented that there was delayed in decision making to transfer the mother immediately to referral facility. A number of social obstacles still persist in the society which largely influenced the family to act in a certain way during a critical situation.

### Issues related to delivery by traditional birth attendant and delivery conduction at home

Home delivery and no-use of trained birth attendance during delivery are major problems. This finding also support previous finding of a national representative study [15]. The social autopsy in the current study also revealed that community people have a high dependency on the traditional birth attendant and village doctor during delivery who are not trained in the management of maternal complications. Maternal death review has been performed in many other countries in similar settings identified delivery by untrained people caused maternal deaths [16–21]. A recent study from Bangladesh also argued that majority of the cases who sough formal care for maternal complications were from informal care facilities [22]. A study conducted in Pakistan has shown that 42 % of deliveries are conducted at home and the majority of the mothers who died did so from haemorrhage and eclampsia. Most of the patients delayed arriving at hospital due to poor decision making made at home with the family [16]. Another study in India illustrated that there are a number of socio-cultural factors due to inadequate knowledge and ignorance which contributed to maternal deaths. This study also showed that 80 % of the mothers who died at home were being delivered by untrained birth attendant [21]. In this study, it was observed that those mother who were suffered from pre-eclampsia or haemorrhage were died at home in majority cases. While as maternal death review (verbal autopsy) findings of Bangladesh shown overall majority deaths occurred in mother at the facility or on the way to facility [5].

### Community delays identified as a barrier

The study also identified that the deceased family were waited until the village doctor or traditional birth attendant spoke about referral as the community has great confidence in village doctors. Moreover, in the majority of cases, it was observed that delayed decision making contributed to the mother’s death and that most of the deaths could have been averted if the mother was treated at the right time. A Mexican study reported that of 19 mothers who died during complications, the family took a day to decide where to go [21]. However, the study also stated that the community had misconceptions about the standard of health care delivery at the hospital and it was this that made them delay their decision [12]. During social autopsy the community mentioned that one of the reasons they delayed the decision to travel to the hospital was because they worried the delivery might be done by caesarean section and that the operation would cause loss of productivity of work of the mother. One study in Ghana has used social autopsy as an umbrella to explore social dilemmas around a death. The study focused on delays in decision making in five deaths and identified social autopsy as an effective method to improve the understanding of the consequences before death [10]. A study in Kenya addressed the reasons for delays including transport, lack of money and how delays in receiving treatment at the health facility were a major contributor to maternal deaths [11].

### Social autopsy in addressing social factors

In this study, social autopsy explored the social errors, barrier and factors related to death, rather than its capability to be a platform to share, discuss among the villagers on the issues related to death. Moreover, the meeting also raised commitment, encouragement and reinforcement of the villagers to decide upon what things they would do to in the future to prevent such events. A review article from India has identified social autopsy as a powerful tool for raising awareness in the community and some of the key decisions taken by a district utilizing social autopsy findings to accelerate and improve the quality of services [12]. This study reflected a number of decisions made by the community to prevent maternal death due to haemorrhage and pre-eclampsia/eclampsia and how lessons learnt from a death event can influence and mobilize an entire community to decide upon better actions to take in the future.

#### Policy issue

Bangladesh is approaching towards achieving sustainable developmental goal by 2030. Health is one of the key priority area for the government, where government is determined to reduce the burden of maternal death to 70/100000 livebirths or below. Identifying the social stigma in the community though social autopsy is a strong supportive document to take action plan at the local level by the health and family planning department and implement [2]. Therefore social autopsies in the current study may argue that awareness among expecting mothers and their family members or decision makers are highly warranted to control maternal death due to known complications. Thus, will help to reduce the burden of mortality. Moreover, sensitization at the community level stimulate the people to increase demand for seeking quality of care from the facility.

#### Strengths and limitations

The main strength of the current study is using 28 social autopsies consists of 761 participants to explore community responses to prevent future maternal deaths. SAs were conducted by highly experienced government health workers within government system involving family decision makers, and community people from all hierarchy in the rural context. Therefore the SAs were concerted efforts between community people, health system persons and researchers, which precisely indicated the system gap to reduce maternal deaths in social perspectives.

The information retrieved in this study is from community where a large group of people shared their notions. Homogeneous character such as the participants could be distant relative or from same ideology, they could be biased by some community leader’s presence are some potential bias of the SAs in the current study [23]. However, those limitations are beyond researchers’ control. Health system workers such as health Inspector, assistant health inspector and family planning inspector were the facilitators and tried to minimize the interpersonal influence by reiterating that the participants should express their views independently. There could be some other methodological issues, beyond researcher’s control. While as the study was first time being conducted and revealed so many important issues for reducing maternal death using SAs.

## Conclusions

Social autopsy of maternal deaths due to haemorrhage and convulsions has been found to act as a catalyst in sensitizing the community to the social causes and errors which led to specific deaths. Using the social autopsy event, the community acknowledge their errors and take corrective measures to prevent future deaths. This powerful commitment has the capacity to influence and transmit the essence of good practice within surrounding communities which could be an effective tool in reducing maternal death in Bangladesh.

## Abbreviations

AB: Animesh Biswas
AH: Abdul Halim
ANC: Antenatal Care
FR: Fazlur Rahman
KD: Koustuv Dalal
MNDR: Maternal and neonatal death review
